# The Simultaneous Removal of Ammonium and Manganese from Surface Water in South China by Manganese Co-Oxide Film

**DOI:** 10.3390/toxics11010022

**Published:** 2022-12-26

**Authors:** Xiangxuan Xing, Tinglin Huang, Ya Cheng, Ruizhu Hu, Gang Wen, Kai Li

**Affiliations:** 1 Key Laboratory of Northwest Water Resource, Environment and Ecology, MOE, Xi’an University of Architecture and Technology, Xi’an 710055, China; 2 Shaanxi Key Laboratory of Environmental Engineering, Xi’an University of Architecture and Technology, Xi’an 710055, China

**Keywords:** surface water, alkalinity, iron–manganese complex oxide film, catalytic oxidation

## Abstract

Exceeding the permitted manganese (Mn^2+^) and ammonium (NH_4_^+^-N) levels is a frequent seasonal occurrence in a water treatment plant in south China. An iron Fe–Mn complex oxide film was found capable of removing more than 95% of Mn^2+^ and NH_4_^+^-N at a water temperature of 20 °C and an alkalinity level of 30 mg/L. It could remove up to 5.5 mg/L of Mn^2+^ and up to 3.5 mg/L of NH_4_^+^-N in a stable manner. Alkalinity is a crucial factor in the removal process. The morphology, elemental composition, and micro-structure of the oxide film were investigated using a scanning electron microscope, an energy-dispersive spectrometer, a Brunauer–Emmett–Teller specific surface-area analyzer, an X-ray diffractometer, and a Fourier-transform infrared spectrometer. The capacity of the Fe–Mn complex oxide film on the surface of the filter medium increased appreciably as its content and specific surface area increased. This research, which provides a theoretical basis for simultaneous manganese and NH_4_^+^-N removal by catalytic oxidation, demonstrates an engineering reference value.

## 1. Introduction

Ammonium (NH_4_^+^-N) and manganese (Mn^2+^) are two common pollutants in water sources, often seen in groundwater and polluted surface water [1]. Different kinds of raw water may contain one substance of either NH_4_^+^-N or manganese, or the two substances may coexist. Drinking water with a high concentration of NH_4_^+^-N, used over an extended period of time, will seriously endanger human health [2]. This is mainly because the excessive intake of NH^4+^ may lead to changes in the acid-base balance of the human body, interfere with glucose tolerance, and reduce the tissue sensitivity of insulin, thus affecting the normal metabolism of the human body [3]. Prolonged consumption of water with high Mn^2+^ content may damage the human central nervous system, leading to fatigue, headaches, memory loss, muscle pain, pneumonia, and even muscle tremors and walking difficulties in severe cases [4]. Therefore, it is necessary to adopt reliable methods to remove NH_4_^+^-N and manganese from raw water.

Conventionally, break-point chlorination [5], the stripping method, ion exchange [6,7], and biological methods [8,9] are used to get rid of NH_4_^+^-N; while pre-oxidation with strong oxidants combined with coagulation, precipitation, and filtration processes [10] are mainly used to remove Mn^2+^. However, for the raw water with NH_4_^+^-N and manganese coexisting, Huang and others found a new way to remove the two pollutants simultaneously in a previous study [11,12]. They discovered the phenomenon of simultaneous removal of NH_4_^+^-N and manganese by chemical catalytic oxidation based on a composite manganese oxide film, which changed the understanding of the leading role of microbial oxidation in removing NH_4_^+^-N. The chemical catalytic oxidation method based on a composite manganese oxide film has opened up a new way, namely an innovative technique to effectively remove NH_4_^+^-N and manganese in raw water. Distinct from conventional processes, it has such advantages as rapid filtration start-up, high pollutant removal efficiency, the absence of by-products, and simple management and operation [13,14]. Guo et al. found that catalytic oxidation was effective in simultaneously removing NH_4_^+^-N and Mn^2+^ from groundwater [12]. When applied to the surface water in north China, which was pretreated without coagulation and precipitation, our catalytic-oxidation filter media, which could simultaneously remove only 1.5 mg/L of NH_4_^+^-N and 0.8 mg/L of Mn^2+^ [15,16], showed a considerably weak ability to remove NH_4_^+^-N and Mn^2+^.

China is a vast country and is usually divided into seven major valleys [17]. The basic physical and chemical properties—such as pH, alkalinity, and turbidity—of both the southern and northern water systems are quite different [18]. In recent years, we have studied the efficiency of iron–manganese complex oxides in the catalytic oxidation of NH_4_^+^-N and Mn^2+^ in the surface water in north China and focused our exploration on the impact of NH_4_^+^-N on Mn^2+^ removal [19]. We take into consideration, however, that the difference in surface-water quality between the north and the south may lead to a difference in the catalytic activity of iron–manganese complex oxides. In addition, a clear knowledge of the NH_4_^+^-N and Mn^2+^ removal efficiency of the iron–manganese complex oxide filter material in southern surface water is the first step to realizing the widespread application of the catalytic oxidation technology in the southern surface-water environment. Therefore, this paper is focused on the application effect of the iron–manganese complex oxide filter material in southern surface water. In order to further improve the technical system of NH_4_^+^-N removal by composite manganese oxide film and optimize the surface-water treatment process, all tests in this study were conducted in south China by using composite manganese oxide filter media to directly filter the source water. The objective of this paper is focused on the following points: (1) the adaptability of the catalytic oxidation filter media to the source water quality; (2) the sustainability of their NH_4_^+^-N and manganese removal effect; and (3) the influence of the pH value and alkalinity on the filter material performance.

## 2. Materials and Methods

### 2.1. Experimental Setup

**Figure 1:**
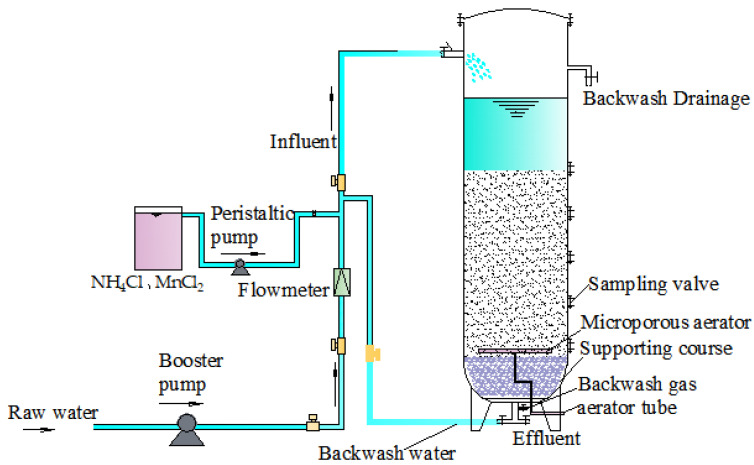
Schematic of the pilot experimental system for the catalytic oxidation of surface water for treatment.

The pilot filter column system (Figure 1) used in this study was located in a surface-water treatment plant. Made of stainless steel, the filter column, with an inner diameter of 400 mm and a total height of 2.2 m, was filled with a filter medium to a height of 100 cm. There was a 15-cm-thick graded gravel layer composed of pebbles at the bottom of the filter column. During the operational process, a hydraulic head of 30–40 cm was maintained above the filter layer. Samples were collected from the filter column at six sites, which were 0, 20, 40, 60, 80, and 100 cm, respectively, below its top surface. The filled filter medium was brought from a groundwater treatment plant in north China. The filter medium, which had a particle size of 0.9–1.2 mm, had black Fe–Mn composite oxides successfully attached onto the surface. The filter column filtered water downward at a rate of 8 m/h. To prevent the filter column from clogging when the water level reached the overflow height, the filter column was flushed with both air and water (for approximately 6 min).

### 2.2. Water-Quality Parameters

The water used in the experiment was obtained from the influent of a surface-water treatment plant in south China, the surface water was filtered directly through the filter column to remove NH_4_^+^-N and Mn^2+^ without any pretreatments. The NH_4_^+^-N and Mn^2+^ in the water originated respectively from artificially added NH_4_Cl and MnCl_2_ chloride solutions. Table 1 summarizes the water-quality parameters of the influent entering the filter column during the experiment.

### 2.3. Experimental Methods

#### 2.3.1. Effectiveness of the Catalytic-oxidation Filter Column in Removing Mn^2+^ and NH_4_^+^-N during Continuous Operation

The surface-water source in south China was treated without coagulation and precipitation pretreatments. The continuous operation lasts about 120 days and can be divided into two stages. At stage I, the concentrations of Mn^2+^ and NH_4_^+^-N in the influent were controlled respectively at 1.5 ± 0.2 and 1.0 ± 0.2 mg/L. This stage lasted about 16 days. At stage II, the average concentrations of NH_4_^+^-N and Mn^2+^ in the influent were controlled at 2.5 and 3.0 mg/L within a certain period of time, respectively. This stage lasted about 104 days.

#### 2.3.2. Effects of Alkalinity on the Mn^2+^ and NH_4_^+^-N Removal Efficiency

The maximum capacity of the oxide film was investigated when the NH_4_^+^-N and Mn^2+^ from the surface-water source were being removed by altering the alkalinity of the raw water and increasing the concentrations of NH_4_^+^-N and Mn^2+^. HCl and NaOH solutions were used to adjust the pH of the influent to 6.51, 7.03, 7.49, and 8.13 during different periods of time to examine the effects of the pH of water on the NH_4_^+^-N and Mn^2+^ removal efficiency.

### 2.4. Analytical Test Methods

Routine indicators were measured using standard methods [20]. The concentration of NH_4_^+^-N was measured using Nessler’s reagent spectrophotometry. Alkalinity was measured using the acid–base indicator titration method. The pH was measured using a Leici PHS-3C pH meter. Turbidity was measured using a Leici WGZ-2000 turbidity meter.

Surface morphology was analyzed using a Quanta FEG 250 scanning electron microscopy (SEM, FEI Quanta 600) system. The main elemental composition of the oxide film was analyzed using an energy-dispersive X-ray spectroscopy (EDS, Oxford INCA/ENERGY- 350) system. The surface-oxide content was determined using the dissolution–reduction method [21]. The specific surface area was measured using the Brunauer–Emmett–Teller method on a Micromeritics ASAP2020 analyzer. The chemical structure of the oxide film was analyzed using a Panalytical Empyrean Xpert Pro MPD X-ray diffractometry (XRD, Ultiman IV) system. The functional-group structure of the oxide film was analyzed using a Fourier-transform infrared spectrometry (FTIR, Nicoli iS50) system. The physical and chemical properties of the oxide film were analyzed based on the pollutant-removal efficiency and the microscopic characterization of the filter medium to determine the mechanism of the change in its activity.

## 3. Results

### 3.1. Effectiveness of the Oxide Film in Removing Mn^2+^and NH_4_^+^-N during Continuous Operation

**Figure 2:**
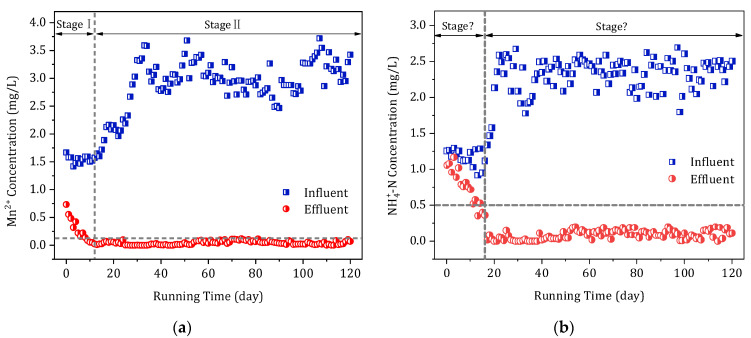
Effectiveness of the oxide film in removing (**a**) Mn^2+^ and (**b**) NH_4_^+^-N during continuous operation.

The temperature of the water during the pilot experiment ranged from 18.0 to 21.0 °C. Figure 2 shows the effectiveness of the oxide film in removing Mn^2+^ and NH_4_^+^-N during continuous operation. Stages I and II refer to the period of the adaption of the filter medium to the water source and to the stable operation period of the filter column, respectively.

An analysis of stage I in Figure 2a revealed that, despite exhibiting some effectiveness in treating Mn^2+^, the filter medium required approximately 8 days of recovery from its inactivity (of some time) to produce an effluent with a concentration of Mn^2+^ below 0.1 mg/L. Subsequently, the filter medium displayed a continuously increasing treatment ability, capable of removing Mn^2+^ completely from the water. A further analysis of stage I in Figure 2b revealed that the effectiveness of the filter column in removing NH_4_^+^-N was similarly unremarkable. After 8 days of operation, the filter column removed NH_4_^+^-N at a rate of approximately 0.5 mg/L.

The concentration of NH_4_^+^-N in the effluent remained below 0.5 mg/L when the operation was conducted until the 14th day. After that, the filter column exhibited a continuously increasing treatment ability and produced an effluent with a concentration of NH_4_^+^-N < 0.1 mg/L. The experimental results for the early-stage adaptation period showed that the oxide film removed Mn^2+^ faster when used to treat the water from the surface-water source and that the oxide film was fully adapted to the local water-quality conditions. Thus, it was able to remove both Mn^2+^ and NH_4_^+^-N in a stable manner after two weeks.

At stage II, the reactivated pilot filter column system ran continuously for 120 days, during which time the concentrations of NH_4_^+^-N and Mn^2+^ in the effluent were monitored. The oxide film removed Mn^2+^ and NH_4_^+^-N in a stable fashion, which could remove more than 95% of these two pollutants simultaneously (Figure 2). In addition, the concentrations of Mn^2+^ and NH_4_^+^-N in the effluent were both lower than those concentrations stipulated regarding the national drinking-water quality standards. The Fe–Mn complex oxides maintained a high catalytic-oxidation capacity for Mn^2+^ and NH_4_^+^-N even if their concentrations in the influent were increased to 3.5 and 2.5 mg/L, respectively. This result is better than expected. Especially when compared with our research on surface-water treatment in north China, iron–manganese complex oxide film can remove only 1.5 mg/L of NH_4_^+^-N and 0.8 mg/L of Mn^2+^ in surface-water treatment in the north. The difference in the water quality between the two regions leads to the clear difference in the catalytic activity of manganese oxide film. In south China, when used to improve the water quality, the oxide film has a better effect than in north China. Therefore, the Fe–Mn composite oxide film exhibited a good adaptability to the water quality of the surface-water source in south China and could effectively and simultaneously remove NH_4_^+^-N and Mn^2+^.

### 3.2. Changes in the Properties of the Oxide Film over Time

#### 3.2.1. Changes in the Content, Specific Surface Area, Pore Size, and Pore Volume of the Oxide Film

The content, specific surface area, pore volume, and pore size of the oxide film on the surface of the filter medium were each measured during the initial-stage operation, during the 15-day operation, and during the 120-day operation (Table 2). The content and specific surface area of the oxide film on the surface of the filter medium increased considerably with the operation time. Up to 120 days, the two indicators mentioned above increased by 57.39% and 156.16%, respectively, as compared with the initial stage of operation. The pore volume of the oxide film also increased, whereas its pore size did not change substantially. The increase in the content and specific surface area of the oxide film may have further enhanced its NH_4_^+^-N and Mn^2+^ removal activity.

#### 3.2.2. Changes in the Surface Morphology of the Oxides

**Figure 3:**
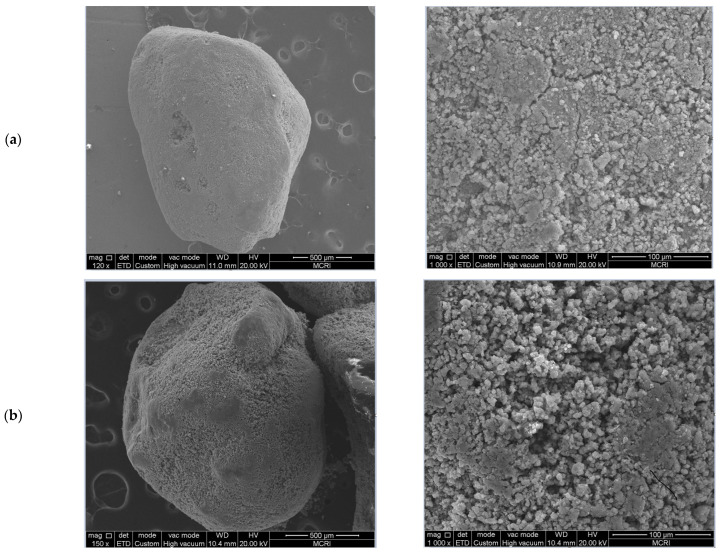

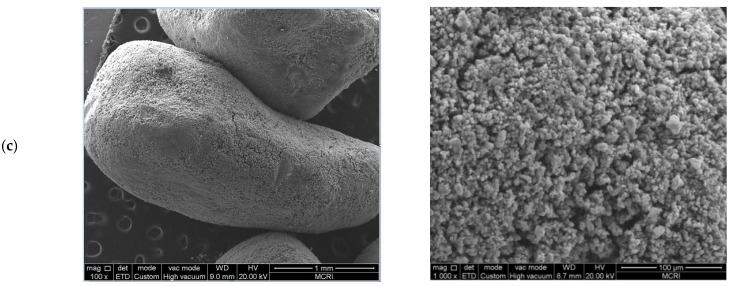
Scanning electron microscopy images of the oxide film. (**a**) Original oxide film, (**b**) after 15 days of operation, and (**c**) after 120 days of operation. The images on the left and right were taken at 100× and 1000× magnification, respectively.

The microscopic surface morphology of the samples of the filter medium was observed using SEM to determine its changes during the continuous operation (Figure 3). Marked morphological changes were found in the filter medium during the operation of the filter column. At the initial stage, the filter medium had a smooth surface with a low oxide content, and the oxide film had a board-like morphology, which was not conducive to the adsorption of NH_4_^+^-N and Mn^2+^ ions. After an extended period of operation, new Fe–Mn composite oxides gradually formed and became attached to the surface of the filter medium during the removal of pollutants. At the later stages, the surface of the filter medium was loose and coarse, and the oxide film featured a sponge-like structure composed of tiny, interconnected micro-lamellar structures. This structure facilitated the contact between the oxide film and the adsorbed pollutants. NH_4_^+^-N and Mn^2+^ were, therefore, removed through catalytic oxidation until the oxide film was matured and, thus, capable of removing NH_4_^+^-N and Mn^2+^ in a stable and continuous manner.

#### 3.2.3. Changes in the Elemental Composition of the Oxide Film

The main elemental composition of the Fe–Mn composite oxides was analyzed using an EDS system (Table 3). The analysis identified Mn, O, Al, Ca, Fe, and Si as the main constituent elements of the filter film on the surface of the filter-medium samples.

The content of Mn^2+^ on the surface of the filter medium was 36.23% in the initial stage of operation of the filter column and then increased to 41.45% and 53.58% after 20 and 100 days of operation, respectively. This indicates an increase of 53.4%, as compared with the initial stage of operation. Moreover, the content of Fe on the surface of the filter medium increased by 150% relative to the initial stage of operation. Therefore, it can be concluded that the Fe–Mn composite oxide film grew substantially during the continuous operation, leading to an increase in its activity. This result can also be corroborated by the aforementioned experimental results for the removal of Mn^2+^ and NH_4_^+^-N.

#### 3.2.4. XRD Analysis of the Oxide Film

**Figure 4:**
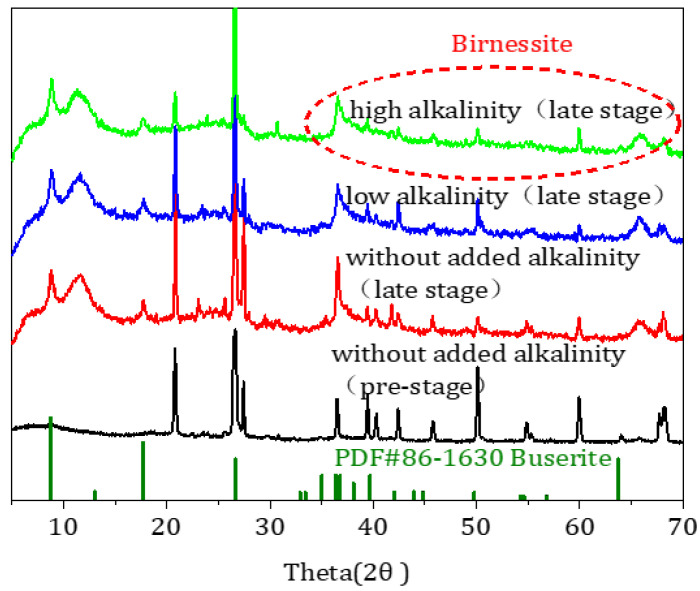
X-ray diffractometry patterns of the filter medium during the operation of the filter column.

By using XRD, the changes in the crystal structure of the oxide film on the surface of the filter medium were analyzed. The XRD patterns generally displayed low peak intensities and broad diffraction peaks, indicating that the Fe–Mn composite oxide samples, at a low degree of crystallinity, could provide a large number of adsorption sites. The XRD pattern of the filter medium changed markedly with the time of operation (Figure 4). It should be noted that SiO_2_ was responsible for the strong peaks at 20.83° and 26.70°. The presence of SiO_2_ can be ascribed to quartz sand, the material used to produce the filter medium. Specifically, compared with the pre-stage operation, the spectrogram at the later stage tends to show more regular peak intensities and broader diffraction peaks. Compared with the previous research results, it was found that the spectrogram of the filter medium at a later stage of operation was consistent with the previous studies, which had a mixed phase structure (birnessite, buserite PDF#86-1630) [22,23]. At the later three stages, the crystal form and phase of the filter media did not show any obvious changes, which indicated that stopping alkalinity after stable operation had no influence on the structure of the Fe–Mn co-oxide film. It also reflected that the crystal-structure stability of Fe–Mn co-oxides was always better.

#### 3.2.5. FTIR Analysis of the Oxide Film

**Figure 5:**
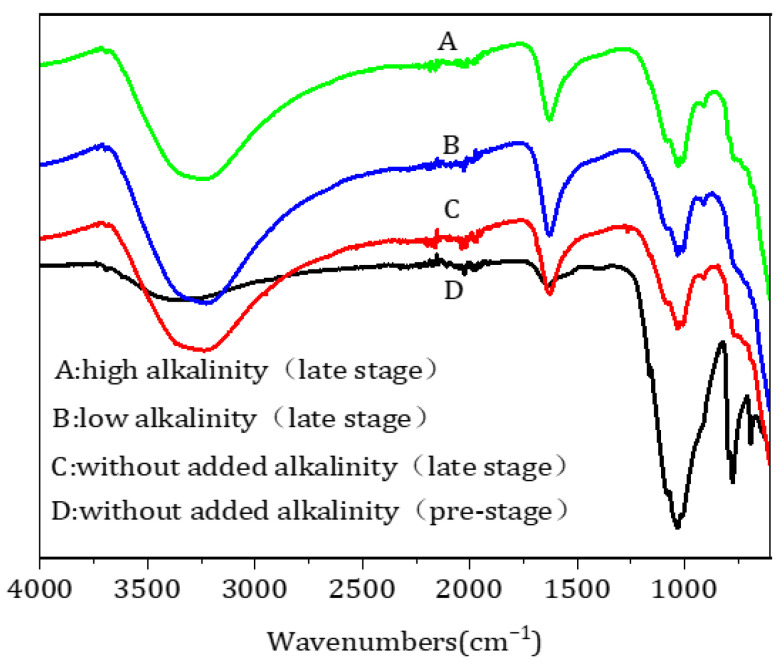
Fourier-transform infrared spectrometry spectra of the filter medium during the operation of the filter column.

FTIR was used to analyze the oxide film on the surface of the filter medium to determine the changes in its functional groups. The filter-medium samples collected in four different periods of operation exhibited three main absorption bands around 3300, 1634, and 1034 cm^–1^ (Figure 5). The absorption band around 3300 cm^–1^ corresponded to the stretching vibrations of water molecules or –OH groups, whereas the –OH functional groups of water molecules adsorbed on the surface of the filter medium were responsible for the absorption band around 1634 cm^–1^ [24,25]. The absorption band near 1041 cm^–1^ was induced by the C–O stretching vibrations of C–O–C functional groups [26]. The intensity of this band decreased with the operation time of the filter medium, suggesting that C–O–C functional groups participated in the reaction during the maturation of the oxide film, whereas the absorption band did not change. Cheng et al. [27] found that Mn^2+^ oxides formed in high-alkalinity environments would lose C–O–C functional groups. The findings from the current study, however, showed that C–O–C functional groups did not change substantially with the time of operation after the oxide film matured.

### 3.3. Analysis of the Effectiveness of the Oxide Film in Removing Mn^2+^ and NH_4_^+^-N at Increased Alkalinity

**Figure 6:**
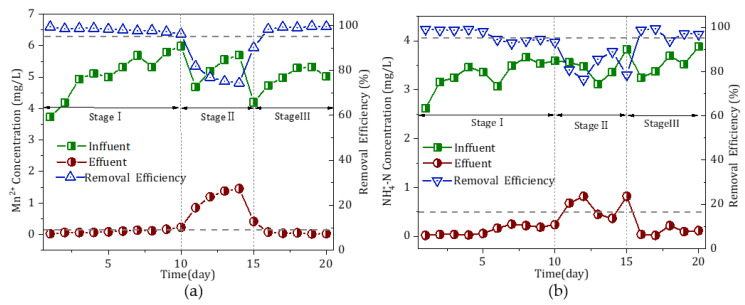
Effects of alkalinity on the effectiveness of the oxide film in removing (**a**) Mn^2+^ and (**b**) NH_4_^+^-N.

Excessively low alkalinity is not conducive to the removal of NH_4_^+^-N and Mn^2+^ from water. The experimental results indicated that NH_4_^+^-N and Mn^2+^, with respective concentrations of 2.5 and 3.5 mg/L, were removed at a low alkalinity level (as low as 30 mg/L). In this study, alkalinity was altered to further enhance the effectiveness of the oxide film in removing NH_4_^+^-N and Mn^2+^ from the surface water. The concentrations of Mn^2+^ and NH_4_^+^-N in the influent were increased to examine the maximum capacity of the oxide film to remove Mn^2+^ and NH_4_^+^-N (Figure 6). Note that alkalinity (in terms of the bicarbonate concentration) was increased to 75 mg/L at stage I, terminated at stage II, and maintained at the same level at stage III as at stage I.

During the operation in which Mn^2+^ was continuously introduced but was not detected in the effluent at stage I, the concentration of Mn^2+^ in the influent was increased from 3.5 to 5.5 mg/L (Figure 6a). The concentration of Mn^2+^ in the effluent remained at approximately 0.1 mg/L, even when the concentration of Mn^2+^ in the influent was increased to 6 mg/L. At stage II, terminating the increase in alkalinity led to a significant increase in the concentration of Mn^2+^ in the effluent. At stage III, the supplementation of alkalinity kept the concentration of Mn^2+^ in the effluent below 0.1 mg/L. At stage I, the concentration of NH_4_^+^-N in the effluent remained below 0.5 mg/L as the concentration of NH_4_^+^-N in the influent was increased from 2.5 to 3.8 mg/L (Figure 6b). The NH_4_^+^-N removal efficiency remained above 95% throughout stage I. At stage II, terminating the increase in alkalinity led to a substantial increase in the concentration of NH_4_^+^-N in the effluent. At stage III, the removal capacity of the oxide film recovered. It can be concluded, therefore, that alkalinity can appreciably enhance the capacity of the active oxide film to remove NH_4_^+^-N and Mn^2+^ from the surface water in south China.

### 3.4. Analysis of the Mechanism by Which the Oxide Film That can Remove Mn^2+^ and NH_4_^+^-N after an Increase of Alkalinity Is to Be Strengthened

Conventional coagulants in surface-water treatment plants contain aluminum (Al^3+^). Consequently, during the filtration of water pretreated through coagulation and precipitation processes in surface-water treatment plants to remove pollutants (e.g., NH_4_^+^-N and Mn^2+^), the residual Al in the water to be purified binds to the surface of the oxide film, resulting in a continuous decrease in the activity of the oxide film. Phosphates are often required to keep the oxide film active. However, the results of the current study showed that when used to directly treat source water, the activity of the oxide film can be maintained for an extended period of time after an adaptation period. Alkalinity can enhance the maximum capacity of the oxide film to remove Mn^2+^ and NH_4_^+^-N. This discovery can directly simplify conventional surface-water treatment processes and facilitate an efficient removal of NH_4_^+^-N and Mn^2+^ from water. Moreover, the investigation in this study was the mechanism of activity of the oxide film in the source water with increased alkalinity.

The removal mechanism of manganese is generally believed to be an auto-catalytic effect of manganese oxides. However, quite properly, there has been no successful exploration till now of its transformation route. Starting from an analysis of the correlation between the removal quantity of manganese ions and the release quantity of hydrogen ions during the process of manganese removal, this paper conducts a further investigation of the mechanism of manganese removal by means of catalytic oxidation.

The overall reaction can be expressed using the following equation:

Mn^2+^ + MeO_x_**•**xH_2_O + (x − 1)/2O_2_ +H _2_O = MeO_x_**•**xH_2_O + 2H^+^, (1)

**Figure 7:**
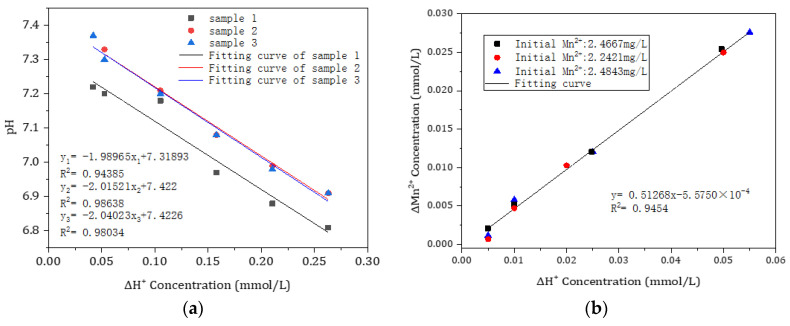
Precision titration experiment. (**a**) Relationship between the increase in the concentration in H^+^ and pH of the raw water, and (**b**) the relationship between the removal of Mn^2+^ and the release of H^+^.

To determine the removal pathway for Mn^2+^, the relationship between the removal of Mn^2+^ and the release of H^+^ was investigated using the following procedure: 50-mL samples of the raw water were used to prepare hydrochloric acid solutions with certain concentrations, and then precision titration was conducted with an acid burette. Changes in the pH of the water samples were measured. A curve plot of the titrated hydrogen ion concentration versus pH was produced for each sample (Figure 7a). These plots were used as a model. Whether or not the decrease in pH due to the formation of H^+^ during the oxidation of Mn^2+^ was consistent with this model was to be testified. The fitted curve of the removal of Mn^2+^ versus the release of H^+^ during the catalytical oxidation process conformed to a first-order linear regression equation with a correlation coefficient (R^2^) of 0.99454 (Figure 7b). Equation (1) shows that the removal of 1 mol of Mn^2+^ is accompanied by the generation of 2 mol of H^+^, which is satisfactorily corroborated by the results of the precision titration experiment. The result of our study suggests that the active oxide film used in the filter medium removed Mn^2+^ through chemical catalytic oxidation.

**Figure 8:**
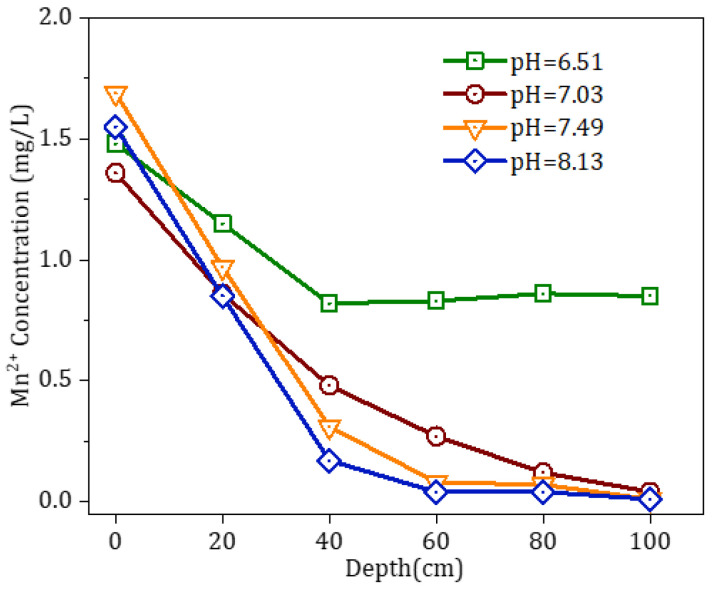
Effects of pH on the removal efficiency of Mn^2+^.

Examined in Figure 8 was the variation in the effectiveness of the oxide film, which was used to remove Mn^2+^ under the condition of different pH values of the influent. When the pH of the influent ranged from 7 to 8, the entire filter medium could remove >1.5 mg/L of Mn^2+^. When the pH of the influent was decreased to 6.5, its middle and lower layers were almost completely ineffective in removing Mn^2+^, resulting in an overall removal efficiency of only approximately 50%, although the upper layer of the filter medium was somewhat effective in removing Mn^2+^. This discovery suggests that a decrease in the pH of the water restricted the capacity of the oxide film to remove Mn^2+^. The measurement of the pH of the effluent of the filter column showed that the pH of the effluent was approximately 6.7 when the pH of the influent was 7. It also suggests that the active oxide film remained effective in removing Mn^2+^ from a neutral or weakly acidic influent from the water source in this region of south China.

In the presence of Fe–Mn complex oxides, NH_4_^+^-N is oxidized into nitrates, generating H^+^. This oxidation process conformed to Equation (2) as follows:

(2) $${\mathrm{NH}}_{4}^{+}+2O_{2}\to {\mathrm{NO}}_{3}^{-}+H_{2}O+2H^{+},$$

The oxidation of NH_4_^+^-N produces H^+^ in the reduction of the pH value and prevents NH_4_^+^-N from being further oxidized. In this study, therefore, increasing alkalinity mitigated the decrease of the pH value and stimulated the oxidation of NH_4_^+^-N. Consequently, adding alkalinity to the influent enhanced the capacity of the filter medium in the filter column to remove NH_4_^+^-N and Mn^2+^.

## 4. Conclusions

In this study, we conducted a series of tests on the ability of composite manganese oxide film to remove manganese and ammonia nitrogen from the raw water in a surface-water plant in south China and analyzed the characteristics of the oxide film before and after operation. The research results are as follows:

(1) Fe–Mn complex oxides showed a high adaptability to the water from a surface-water source in a certain region of south China and displayed a continuously stable capacity to remove NH_4_^+^-N and Mn^2+^ after two weeks of operation. The concentrations of NH_4_^+^-N and Mn^2+^ in the effluent met the requirements stipulated in the drinking water standards in a stable manner when the concentrations of NH_4_^+^-N and Mn^2+^ in the influent reached 2.5 and 3.5 mg/L, respectively.

(2) The active oxide film remained effective in removing Mn^2+^ from a neutral or even weakly acidic influent. The results of the precision titration experiment showed that the active oxide film in the filter medium removed Mn^2+^ through chemical catalytic oxidation.

(3) An increase in alkalinity can help realize the maximum capacity of the active oxide film to remove NH_4_^+^-N and Mn^2+^ up to 3.5 and 5.5 mg/L, respectively, in south China. This achieves a much better result than that in north China.

(4) Analytical test results achieved with the help of SEM, EDS, and XRD revealed that the oxide film underwent some changes in its surface morphology when it was used to remove Mn^2+^ and NH_4_^+^-N from the surface water over an extended period of time, but its microstructure did not change at a fundamental level. The pollutant removal activity of the oxide film on the surface of the filter medium increased somewhat as its content and specific surface area increased.

(5) The expansion of the application scenario of this technology is of great significance for improving the quality of surface water which contains manganese and ammonia nitrogen. This improvement exceeds the standard in southern China and ensures the safety of people’s drinking water.

## Figures and Tables

**Table 1:** Water-quality parameters of the influent entering the filter column.

	Water Quality Indications	Unit	Value
1	T	℃	18–21
2	DO	mg·L^–1^	4.3–6.9
3	pH	-	7.04–7.50
4	Turbidity	NTU	1.17–2.69
5	COD_Mn_	mg·L^–1^	1.07–1.94
6	TP	µg·L^–1^	<15
7	Total dissolved solids	mg·L^–1^	40–50
8	Alkalinity	mg·L^–1^(expressed in CaCO_3_ equivalent)	30–40
9	Total aluminum	μg·L^–1^	<30

**Table 2:** Content, specific surface area, pore volume, and pore size of the oxide film on the surface of the filter medium.

No	Operation Time (day)	Specific Surface Area (m^2^/g)	Pore Volume (cm^3^/g)	Pore Size (nm)	Surface Oxide Content (mg/g)
1	Initial stage of operation	1.15	0.0038	13.86	12.66
2	15	1.39	0.0042	12.33	19.48
3	120	1.81	0.0049	14.00	32.43

**Table 3:** Changes in the main elemental composition of the filter medium containing Fe–Mn composite oxides.

	O (%)	Al (%)	Si (%)	K (%)	Ca (%)	Mn (%)	Fe (%)
Initial stage of operation	54.45	4.83	2.16	0.10	1.17	36.23	1.06
After 20 days of operation	49.08	4.95	1.17	0.42	1.58	41.45	1.35
After 100 days of operation	42.13	0.15	0.92	0	0.63	53.58	2.65

## Data Availability

The datasets generated and/or analysed during the current study are available from the corresponding author on reasonable request.
